# Role of DNA methylation in the association of lung function with body mass index: a two-step epigenetic Mendelian randomisation study

**DOI:** 10.1186/s12890-020-01212-9

**Published:** 2020-06-16

**Authors:** André F. S. Amaral, Medea Imboden, Matthias Wielscher, Faisal I. Rezwan, Cosetta Minelli, Judith Garcia-Aymerich, Gabriela P. Peralta, Juha Auvinen, Ayoung Jeong, Emmanuel Schaffner, Anna Beckmeyer-Borowko, John W. Holloway, Marjo-Riitta Jarvelin, Nicole M. Probst-Hensch, Deborah L. Jarvis, for the ALEC consortium

**Affiliations:** 1 National Heart and Lung Institute, Imperial College London, London, UK; 2 Swiss Tropical and Public Health Institute, Basel, Switzerland; 3 University of Basel, Basel, Switzerland; 4 Epidemiology and Biostatistics, School of Public Health, Imperial College London, London, UK; 5 Human Development and Health, Faculty of Medicine, University of Southampton, Southampton, UK; 6 ISGlobal, Barcelona, Spain; 7 Universitat Pompeu Fabra (UPF), Barcelona, Spain; 8 CIBER Epidemiología y Salud Pública (CIBERESP), Barcelona, Spain; 9 Faculty of Medicine, University of Oulu, Oulu, Finland

**Keywords:** Body mass index, Lung function, DNA methylation, Effect mediation, Mendelian randomisation

## Abstract

**Background:**

Low lung function has been associated with increased body mass index (BMI). The aim of this study was to investigate whether the effect of BMI on lung function is mediated by DNA methylation.

**Methods:**

We used individual data from 285,495 participants in four population-based cohorts: the European Community Respiratory Health Survey, the Northern Finland Birth Cohort 1966, the Swiss Study on Air Pollution and Lung Disease in Adults, and the UK Biobank. We carried out Mendelian randomisation (MR) analyses in two steps using a two-sample approach with SNPs as instrumental variables (IVs) in each step. In step 1 MR, we estimated the causal effect of BMI on peripheral blood DNA methylation (measured at genome-wide level) using 95 BMI-associated SNPs as IVs. In step 2 MR, we estimated the causal effect of DNA methylation on FEV_1_, FVC, and FEV_1_/FVC using two SNPs acting as methQTLs occurring close (in cis) to CpGs identified in the first step. These analyses were conducted after exclusion of weak IVs (F statistic < 10) and MR estimates were derived using the Wald ratio, with standard error from the delta method. Individuals whose data were used in step 1 were not included in step 2.

**Results:**

In step 1, we found that BMI might have a small causal effect on DNA methylation levels (less than 1% change in methylation per 1 kg/m2 increase in BMI) at two CpGs (cg09046979 and cg12580248). In step 2, we found no evidence of a causal effect of DNA methylation at cg09046979 on lung function. We could not estimate the causal effect of DNA methylation at cg12580248 on lung function as we could not find publicly available data on the association of this CpG with SNPs.

**Conclusions:**

To our knowledge, this is the first paper to report the use of a two-step MR approach to assess the role of DNA methylation in mediating the effect of a non-genetic factor on lung function. Our findings do not support a mediating effect of DNA methylation in the association of lung function with BMI.

## Background

There are several cross-sectional studies showing lower forced respiratory volumes (FEV_1_ and FVC) in those who are overweight and obese. Evidence on the association between obesity and FEV_1_/FVC is less clear [1]. Longitudinal studies also suggest that an increase in body mass index (BMI) is associated with increased lung function decline. An increase in BMI among overweight and obese people has been associated with greater than average decline of FEV_1_ and FVC [2, 3]. There is always a risk that such associations may be confounded, but a large Mendelian randomisation (MR) study has shown that increasing BMI leads to the decline in both FEV_1_ and FVC [4], suggesting that this association is causal.

The underlying mechanisms for this association are unclear with some hypothesising that it reflects pulmonary biological responses to obesity and its related pro-inflammatory status, and others suggesting it reflects thoracic compression (i.e., a mechanical effect) [5, 6]. As both differences in BMI and lung function have been associated with DNA methylation levels [7–10], we hypothesised that association of lung function with BMI is in part mediated by DNA methylation.

A new method using MR to investigate the mediating effect of DNA methylation on the association of risk factors and health outcomes has recently been described [11, 12]. This two-step epigenetic MR approach relies on the use of genetic variants potentially controlling DNA methylation. In the first step, a single nucleotide polymorphism (SNP), or group of SNPs, that proxies for the risk factor of interest (here BMI) is used to assess the causal relationship between the risk factor and DNA methylation. If this association is confirmed, in the second step, a SNP that proxies for methylation levels at the site modified by the risk factor is used to interrogate the causal relationship between DNA methylation and the main outcome (here lung function). We applied this technique to investigate the mediating role of DNA methylation in the association of lung function with BMI in European cohorts, after using a conventional non-MR approach.

## Methods

### Conventional (non-MR) analysis

We assessed the cross-sectional association of lung function (FEV_1_, FVC, FEV_1_/FVC) with BMI in participants of the European Community Respiratory Health Survey (ECRHS, *n* = 470), the Northern Finland Birth Cohort 1966 (NFBC1966, *n* = 681), and the Swiss Study on Air Pollution and Lung Disease in Adults (SAPALDIA, *n* = 962) (see Supplementary File 1 for details) who also had information on peripheral blood DNA methylation. This analysis was carried out using linear regression models adjusted for centre, sex, age, height, sex-age interaction, sex-height interaction, educational level, smoking status, and pack-years of smoking. We estimated the association of each lung function parameter with BMI for each cohort, and then combined them in a random effects meta-analysis.

### Two-step MR

We carried out MR analyses in two steps using a two-sample approach for summary data with SNPs as instruments in each step. To assess the strength of each SNP as instrument, we calculated the F statistic [13], and to avoid bias due to use of weak instruments, we included in the MR analyses only SNPs with an F statistic equal to or greater than 10 [14]. Individual-level data used in these analyses come from four cohorts: ECRHS, NFBC1966, SAPALDIA, and UK Biobank (see Supplementary File 1 for details). Analyses were conducted using R v.3.3.2 [15].

### First-step MR: examining the causal effect of BMI on DNA methylation

#### SNP-BMI association estimates

**Fig. 1:**
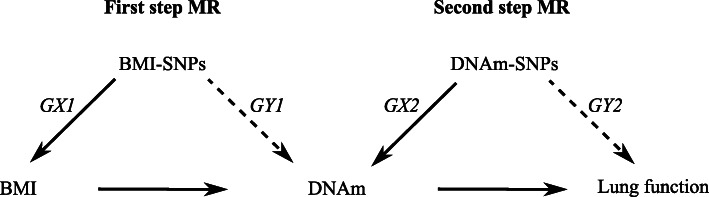
Flow diagram of the 2-step epigenetic Mendelian randomisation analysis

A recent published genome-wide association meta-analysis of 125 studies on 339,224 participants reported the association of BMI with 97 SNPs (accounting for an estimated 2.7% of the variability of BMI in the population) [16]. We extracted their effect estimates and standard errors and used these SNPs as instruments in the first step MR (Fig. 1: GX1).

#### SNP-DNA methylation estimates

We created a weighted genetic risk score (wGRS) for BMI from the 97 SNPs for 470 participants from the ECRHS and 681 participants from the NFBC1966. The wGRS was the sum of the products of the effect allele dosage at each of the 97 SNPs and their corresponding beta coefficients [16]. As a screening stage we used linear regression to measure the effect of the wGRS on DNA methylation (Table 1). CpGs associated with BMI-wGRS at *P* < 10^− 7^ were then examined to assess their association with individual SNPs (Fig. 1: GY1) contributing to the wGRS. Replication of the identified associations was sought in the SAPALDIA cohort (*n* = 906) (Table 1). These analyses were adjusted for ancestry principal components. Participants included in this analysis are those included in the conventional (non-MR) analysis, except for 56 participants from SAPALDIA for whom there were no allele dosage data.

**Table 1:** Body mass index and lung function of study populations included in the genotype-outcome association analyses

MR step	Study	N	Age (years), median (IQR)	BMI (kg/m^2^), median (IQR)	FEV_1_ (L), median (IQR)	FVC (L), median (IQR)	FEV_1_/FVC (%),median (IQR)
1st	ECRHS	470	55 (49–60)	26.0 (23.3–29.3)	2.9 (2.4–3.5)	3.8 (3.2–4.6)	76 (72–80)
	NFBC1966	681	46 (46–47)	25.8 (23.3–29.1)	3.4 (2.9–4.0)	4.4 (3.8–5.2)	77 (74–81)
	SAPALDIA	906	58 (50–68)	25.9 (23.3–29.3)	2.9 (2.4–3.5)	4.0 (3.3–4.8)	74 (69–78)
2nd	ECRHS	773	54 (48–60)	26.3 (23.8–29.8)	2.9 (2.5–3.5)	3.9 (3.3–4.7)	76 (72–80)
	NFBC1966	4501	47 (46–47)	26.1 (23.5–29.4)	3.3 (2.9–3.9)	4.3 (3.7–5.1)	78 (74–82)
	SAPALDIA	2303	60 (50–67)	25.7 (23.1–28.6)	3.0 (2.5–3.6)	4.1 (3.4–4.9)	74 (69–78)
	UK Biobank	275,861	58 (50–63)	26.6 (24.1–29.7)	2.7 (2.3–3.3)	3.6 (3.0–4.3)	77 (73–80)

*MR* Mendelian randomisation. *IQR* Interquartile range. *BMI* Body mass index. *FEV*_1_ Forced expiratory volume in one second. *FVC* Forced vital capacity

#### BMI-DNA methylation estimates: MR analysis

To estimate the causal effect of BMI on DNA methylation levels, we derived MR estimates for each of the 95 SNPs with an F statistic equal to or greater than 10 (Table E1 in Supplementary File 1), using the Wald estimator (ratio of the genotype-outcome regression coefficient to the genotype-exposure regression coefficient), with standard error derived using the delta method [17]. The individual MR estimates were combined using inverse-variance weighted (IVW) fixed-effect meta-analysis [18]. To investigate the robustness of the MR findings to pleiotropy, we used the following methods: 1) IVW random-effects meta-analysis [19]; 2) Egger regression with penalized weights [20]; and 3) weighted median analysis [21].

### Second-step MR: examining the causal effect of DNA methylation on lung function

#### DNA methylation-cis-SNP association estimates

Using the publicly available mQTL database (http://mqtldb.org; accessed on 1 December 2017), which contains the associations of peripheral blood DNA methylation with SNPs as observed in the ALSPAC-ARIES project, we identified SNPs associated with the CpGs discovered in step 1 (*p* < 10^− 7^) and located within 1 Mb either side of the CpG (*cis*-SNPs) [22] (Fig. 1: GX2). We selected independent *cis*-SNPs, after linkage disequilibrium (LD) clumping (‘clump_data’ function from R package ‘TwoSampleMR’), as the instruments for the DNA methylation of interest. The regression coefficient and standard error for the *cis*-SNP-methylation association were used in the MR analysis.

#### Cis-SNP-lung function association estimates

We regressed lung function parameters (FEV_1_, FVC, FEV_1_/FVC) on the *cis*-SNPs (Fig. 1: GY2) in the ECRHS (*n* = 773), NFBC1966 (*n* = 4501), SAPALDIA (*n* = 2303), and UK Biobank (*n* = 275,861) cohorts (Table 1). The analysis was adjusted for ancestry principal components and did not include data from participants included in step one. As a sex difference in the association of lung function with BMI has been reported [2], FEV_1_, FVC and FEV_1_/FVC were adjusted for sex.

#### DNA methylation-lung function estimates: MR analysis

To estimate the causal effect of DNA methylation on lung function, we derived MR estimates for each SNP with an F statistic equal to or greater than 10 using the Wald estimator, with standard error derived using the delta method [17].

## Results

A description of the BMI and lung function parameters of the participants in the several cohorts included in this analysis is presented in Table 1. On average, BMI was similar across cohorts and lung volumes were higher in NFBC1966 and SAPALDIA.

### Conventional (non-Mendelian randomisation) analysis

**Fig. 2:**
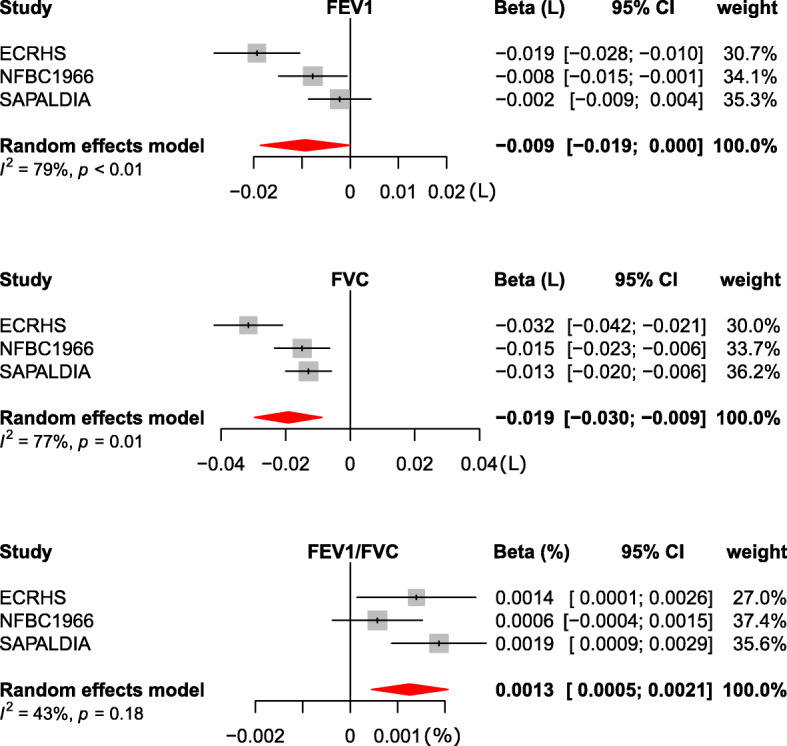
Association of lung function with body mass index: non-Mendelian randomisation approach

A higher BMI was associated with lower FEV_1_ (beta coefficient = − 0.009, 95% CI − 0.019 to 0.0) and FVC (beta coefficient = − 0.19, 95% CI − 0.03 to − 0.009) among participants of ECRHS, NFBC and SAPALDIA who had DNA methylation. The FEV_1_/FVC ratio was positively associated with BMI (beta coefficient = 0.0013, 95% CI 0.0005 to 0.002) (Fig. 2).

### First-step MR: examining the causal effect of BMI on DNA methylation

**Fig. 3:**
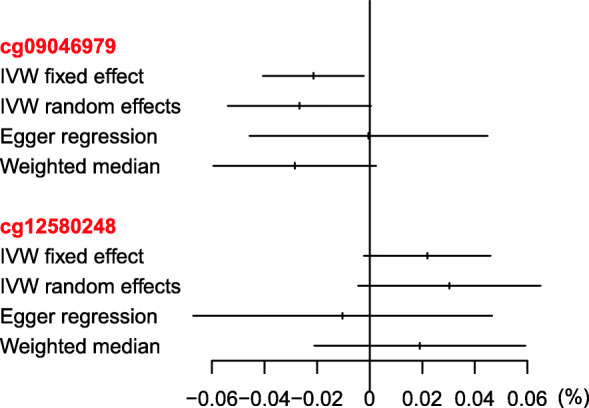
Mendelian randomisation estimates of the causal effect of body mass index on DNA methylation using methods robust to pleiotropy

The genotype-BMI association estimates for the 97 SNPs used as instrumental variables for BMI are presented in Table E1 (see Supplementary File 1). In the screening stage, the 97-SNP wGRS was associated with two CpGs, one in *SBK1* (cg09046979) and one in *NPIPB11* (cg12580248) (Table E2 in Supplementary File 1). MR estimates, from IVW fixed effect meta-analysis, for the effect of BMI on these two CpGs are presented in Table 2. A 1-unit increase in BMI was responsible for less than 1% change in methylation at either of the CpGs. The effect of BMI on cg09046979 was statistically significant in ECRHS and NFBC1966, but not in SAPALDIA. The effect of BMI on cg12580248 was not statistically significant in ECRHS and NFBC1966 and could not be assessed in SAPALDIA as a probe for this CpG is not present in the methylation chip used in SAPALDIA. Results from the IVW random effects meta-analysis, Egger regression and weighted median analysis were consistent with those of the IVW fixed effect meta-analysis (Fig. 3).

**Table 2:** Step 1: IVW fixed-effect MR estimates of the causal effect of BMI on CpG methylation

			ECRHS + NFBC (***n*** = 1151)			SAPALDIA (***n*** = 906)		
CpG	Gene	Chromosomal position	MR estimate (SE)^a^	***P***	I^2b^	MR estimate (SE)^a^	***P***	I^2b^
cg09046979	*SBK1*	16:28333134	−0.021 (0.010)	0.03	41%	0.007 (0.015)	0.6	39%
cg12580248	*NPIPB11*	16:29412940	0.022 (0.012)	0.08	43%	–	–	–

^a^Change in methylation (unit is %) per one-unit increase in BMI (kg/m^2^); *MR* Mendelian randomisation; *SE* Standard error. ^b^Between-instrument heterogeneity

### Second-step MR: examining the causal effect of DNA methylation on lung function

Using the mQTLdb and after LD clumping, we found that methylation at CpG cg09046979 had been associated with two independent SNPs (rs9938394 and rs9939450) occurring within 1 Mb either side of the probe (cis-SNPs). We could not find publicly available information from independent cohorts on genotype-DNA methylation associations where the Infinium MethylationEPIC BeadChip was used to measure levels of methylation. The genotype-DNA methylation association estimates for the two SNPs, which were used as instrumental variables for DNA methylation, are presented in Table E3 (see Supplementary File 1).

MR estimates for the effect of DNA methylation, at cg09046979, on FEV_1_, FVC and the FEV_1_/FVC ratio are presented in Table 3. There was no evidence of an association between methylation levels at this site and lung function.

**Table 3:** Step 2: IVW fixed-effect MR estimates of the causal effect of DNA methylation on lung function

CpG	Gene	Lung function	MR estimate (SE)^a^ (ECRHS + NFBC + SAPALDIA + UK Biobank; ***n*** = 283,476)	***P***
cg09046979	*SBK1*	FEV_1_	−0.0003 (0.003)	0.89
		FVC	−0.001 (0.005)	0.75
		FEV_1_/FVC	−0.0002 (0.003)	0.93

^a^MR estimates (S.E.) represent the change in lung function (L) per methylation proportion (unit is %)

## Discussion

The findings of this 2-step epigenetic MR study suggest a small causal effect of BMI on DNA methylation at one or two CpGs, but also suggest that these are unlikely to exert a causal effect on lung function.

As this is a multicentre study across several countries, confounding due to population stratification is possible. However, most study participants were of European descent and ancestry principal components were included in the analyses. There is always concern within an MR study that pleiotropy (when an SNP affects several phenotypes related to the outcome [23], in this case, DNA methylation in the first step MR and lung function in the second step MR) may exist and there was some evidence of heterogeneity (I^2^ of 41% for cg09046979 and 43% for cg12580248) suggestive of pleiotropy. However, we used methods that are robust to pleiotropy (i.e. IVW random effects, Egger regression, and weighted median) and found results to be consistent with those of the IVW fixed effect analysis. The sample size of the first step MR was limited by the relatively small number of ECRHS, NFBC1966 and SAPALDIA participants with available data on DNA methylation, which may have reduced the chances of identifying causal associations of BMI with DNA methylation. Despite the very large sample size in the second step MR, our capacity to fully explore our findings for one of the CpGs was limited by the lack of information on associations between SNPs and CpG methylation assessed using the EPIC (850 K) chip from independent studies. All studies that utilise peripheral blood DNA methylation data to explore associations of lifestyle and environmental factors with organ specific abnormalities are limited by the lack of consistent clear evidence that DNA methylation in peripheral blood reflects well what is going on in the relevant disease tissue. Although some concordance in DNA methylation levels between blood and lung tissue has been reported [24] as well as for BMI related blood methylation with that in adipose tissue [25], some argue this is unlikely to be common [26]. As variation in DNA methylation levels across the epigenome is often tissue-specific [27], we cannot for sure say that the association of lung function with BMI is not mediated by DNA methylation within lung tissue.

## Conclusion

In conclusion, our findings do not support a mediating effect of peripheral blood DNA methylation in the association of lung function with BMI.

## Supplementary information

**Additional file 1.** Supplementary File 1.

## Data Availability

Access to individual level data is restricted. However, data requests for sound research proposals will be addressed. For ECRHS data, contact DLJ (d.jarvis@imperial.ac.uk). For NFBC1966 data, contact MRJ (m.jarvelin@imperial.ac.uk). For SAPALDIA data, contact NMPH (nicole.probst@swisstph.ch). For UK Biobank data, request should be made through www.ukbiobank.ac.uk.

## Abbreviations

BMI: Body mass index
CpG: 5′-C-phosphate-G-3
DNA: Deoxyribonucleic acid
ECRHS: European community respiratory health survey
FEV_1_: Forced expiratory volume in one second
FVC: Forced vital capacity
IQR: Interquartile range
IV: Instrumental variable
IVW: Inverse-variance weighted
LD: Linkage disequilibrium
methQTL: Methylation quantitative trait locus
MR: Mendelian randomisation
NFBC: Northern Finland birth cohort
SAPALDIA: Swiss study on air pollution and lung disease in adults
SNP: Single nucleotide polymorphism
wGRS: Weighted genetic risk score
