# Supplementary material for: Role of DNA methylation in the association of lung function with body mass index: a two-step epigenetic Mendelian randomisation study
Source: BMC Pulm Med. 2020 Jun 16;20:171. doi: 10.1186/s12890-020-01212-9 (PMC7298775; doi:10.1186/s12890-020-01212-9)
Supplement: Supplementary file 1 — Additional file 1. Supplementary File 1. [file 12890_2020_1212_MOESM1_ESM.docx]

**Supplementary File 1**

**Title:** Role of DNA methylation in the association of lung function with body mass index: A two-step epigenetic Mendelian randomisation study

**Authors:** André F. S. Amaral^1*^, Medea Imboden^2,3*^, Matthias Wielscher^4*^, Faisal I. Rezwan^5*^, Cosetta Minelli^1^, Judith Garcia-Aymerich^6^, Gabriela P. Peralta^6^, Juha Auvinen^7^, Ayoung Jeong^2,3^, Emmanuel Schaffner^2,3^, Anna Beckmeyer-Borowko^2,3^, John W. Holloway^5†^, Marjo-Riitta Jarvelin^4,7†^, Nicole M. Probst-Hensch^2,3†^, Deborah L. Jarvis^1†^, for the ALEC consortium

*co-first authors; †co-last authors

**Affiliations:** ^1^National Heart and Lung Institute, Imperial College London, London, UK; ^2^Swiss Tropical and Public Health Institute, Basel, Switzerland; ^3^University of Basel, Switzerland; ^4^Epidemiology and Biostatistics, School of Public Health, Imperial College London, London, UK; ^5^Human Development and Health, Faculty of Medicine, University of Southampton, Southampton, UK; ^6^ISGlobal, Barcelona, Spain; ^7^Faculty of Medicine, University of Oulu, Oulu, Finland.

**Corresponding author’s contact details:** André F. S. Amaral; National Heart and Lung Institute, Imperial College London, Emmanuel Kaye Building, 1B Manresa Road - London SW3 6LR (UK); Tel: +44 (0) 207 594 7940; Email: [a.amaral@imperial.ac.uk](mailto:a.amaral@imperial.ac.uk)

**Study populations**

*European Community Respiratory Health Survey (ECRHS)*

The European Community Respiratory Health Survey is an international multicentre cohort study designed to assess the prevalence of asthma and allergic disease and identify their risk factors (1). Young adults of European descent were randomly recruited from community-based sampling frames in the ECRHS I (1991-1993) and followed up twice in the 20 years after the first assessment (ECRHS II: 1998-2002; ECRHS III: 2008-2013). For full protocols, see http://www.ecrhs.org.

*Northern Finland Birth Cohort (NFBC) 1966*

The Northern Finland Birth Cohort 1966 is a population-based sample of all live births in 1966 (n = 12,058) in the provinces of Oulu and Lapland in Finland. Women with expected delivery dates in 1966 were recruited through maternity health centres (2). In 1997, at offspring age of 31 years, all cohort participants with known addresses were sent a postal questionnaire on health and lifestyle and those living in Northern Finland or Helsinki area were invited to a clinical examination which included blood sampling. In 2012, all individuals with known address in Finland were sent postal questionnaires and an invitation for clinical examination. This longitudinal, epidemiological research program is maintained within the Department of Health Sciences, Faculty of Medicine, University of Oulu, Finland (http://www.oulu.fi/nfbc).

*Swiss Study on Air Pollution Heart and Lung Disease in Adults (SAPALDIA)*

SAPALDIA began in 1991 to specifically study the air pollution impact on respiratory health (3). It is a cohort of adults aged 18 to 60 years from population registries in eight communities in Switzerland, representing the three largest language groups (German, French, Italian) as well as different levels of air pollution and degrees of urbanization. Participants underwent spirometry and answered a detailed questionnaire on respiratory health, allergies, smoking history, and lifestyle factors at baseline (year 1991) and first and second follow-up (years 2002 and 2011) examinations. Study directorate: NM Probst-Hensch (PI; e/g); T Rochat (p), C Schindler (s), N Künzli (e/exp), JM Gaspoz (c). Scientific team: JC Barthélémy (c), W Berger (g), R Bettschart (p), A Bircher (a), C Brombach (n), PO Bridevaux (p), L Burdet (p), Felber Dietrich D (e), M Frey (p), U Frey (pd), MW Gerbase (p), D Gold (e), E de Groot (c), W Karrer (p), F Kronenberg (g), B Martin (pa), A Mehta (e), D Miedinger (o), M Pons (p), F Roche (c), T Rothe (p), P Schmid-Grendelmeyer (a), D Stolz (p), A Schmidt-Trucksäss (pa), J Schwartz (e), A Turk (p), A von Eckardstein (cc), E Zemp Stutz (e). Scientific team at coordinating centers: M Adam (e), I Aguilera (exp), S Brunner (s), D Carballo (c), S Caviezel (pa), I Curjuric (e), A Di Pascale (s), J Dratva (e), R Ducret (s), E Dupuis Lozeron (s), M Eeftens (exp), I Eze (e), E Fischer (g), M Foraster (e), M Germond (s), L Grize (s), S Hansen (e), A Hensel (s), M Imboden (g), A Ineichen (exp), A Jeong (g), D Keidel (s), A Kumar (g), N Maire (s), A Mehta (e), R Meier (exp), E Schaffner (s), T Schikowski (e), M Tsai (exp). (a) allergology, (c) cardiology, (cc) clinical chemistry, (e) epidemiology, (exp) exposure, (g) genetic and molecular biology, (m) meteorology, (n) nutrition, (o) occupational health, (p) pneumology, (pa) physical activity, (pd) pediatrics, (s) statistics.

*UK Biobank*

The UK Biobank is a population-based study of adults aged 40–69 years recruited from 22 centres across England, Wales, and Scotland. Recruitment relied on invitations sent by mail to both women and men living within 10 miles (16.1 km) of the testing centre. Between 2006 and 2010, 502,628 participants completed a touch screen questionnaire to provide information on their lifestyle as well as their medical and family history, and they underwent clinical assessment including spirometry (4).

**DNA methylation measurement and adjustment for technical factors**

Samples of peripheral blood were collected in ECRHS, NFBC and SAPALDIA using standard operating procedures at two time points on two consecutive follow-up surveys from which DNA was extracted. Data in this study are from the most recent survey. Genome-wide DNA methylation typing including DNA bisulphite conversion was performed at the Wellcome Trust Centre for Human Genetics (Oxford, UK). Quantification of DNA methylation across the genome was carried out using the Illumina® Infinium Human Methylation technology (EPIC/850k BeadChip for ECRHS and NFBC; 450k BeadChip for SAPALDIA).

The quality control processing of the DNA methylaton data used in this study has been published elsewhere (5). Briefly, we performed the methylation data processing within each cohort separately, using R packages minfi (6), RnBeads (7) and CPACOR (8). After standard QC cleaning steps regarding duplicates, sex inconsistency, low sample quality (sample call rate >95%) for all three cohorts, ECRHS additionally excluded outlier samples beyond 1.5 interquartile range (IQR) and NFBC1966 excluded samples with their first principal component score of the DNA methylation values outside the interval given by the mean +/- 4SD. For technical bias correction, the first 30 principal components derived from the control probes were used.

**References**

1. Burney PG, Luczynska C, Chinn S, Jarvis D. The European Community Respiratory Health Survey. *The European respiratory journal : official journal of the European Society for Clinical Respiratory Physiology* 1994; 7: 954-960.

2. Rantakallio P. The longitudinal study of the northern Finland birth cohort of 1966. *Paediatr Perinat Epidemiol* 1988; 2: 59-88.

3. Ackermann-Liebrich U, Kuna-Dibbert B, Probst-Hensch NM, Schindler C, Dietrich DF, Stutz EZ, Bayer-Oglesby L, Baum F, Brändli O, Brutsche M, Downs SH, Keidel D, Gerbase MW, Imboden M, Keller R, Knöpfli B, Künzli N, Nicod L, Pons M, Staedele P, Tschopp J-M, Zellweger J-P, Leuenberger P. Follow-up of the Swiss Cohort Study on Air Pollution and Lung Diseases in Adults (SAPALDIA 2) 1991–2003: methods and characterization of participants. *Sozial- und Präventivmedizin SPM* 2005; 50: 245-263.

4. Sudlow C, Gallacher J, Allen N, Beral V, Burton P, Danesh J, Downey P, Elliott P, Green J, Landray M, Liu B, Matthews P, Ong G, Pell J, Silman A, Young A, Sprosen T, Peakman T, Collins R. UK biobank: an open access resource for identifying the causes of a wide range of complex diseases of middle and old age. *PLoS Med* 2015; 12: e1001779.

5. Imboden M, Wielscher M, Rezwan FI, Amaral AFS, Schaffner E, Jeong A, Beckmeyer-Borowko A, Harris SE, Starr JM, Deary IJ, Flexeder C, Waldenberger M, Peters A, Schulz H, Chen S, Sunny SK, Karmaus WJJ, Jiang Y, Erhart G, Kronenberg F, Arathimos R, Sharp GC, Henderson AJ, Fu Y, Piirila P, Pietilainen KH, Ollikainen M, Johansson A, Gyllensten U, de Vries M, van der Plaat DA, de Jong K, Boezen HM, Hall IP, Tobin MD, Jarvelin MR, Holloway JW, Jarvis D, Probst-Hensch NM. Epigenome-wide association study of lung function level and its change. *The European respiratory journal : official journal of the European Society for Clinical Respiratory Physiology* 2019.

6. Aryee MJ, Jaffe AE, Corrada-Bravo H, Ladd-Acosta C, Feinberg AP, Hansen KD, Irizarry RA. Minfi: a flexible and comprehensive Bioconductor package for the analysis of Infinium DNA methylation microarrays. *Bioinformatics* 2014; 30: 1363-1369.

7. Assenov Y, Muller F, Lutsik P, Walter J, Lengauer T, Bock C. Comprehensive analysis of DNA methylation data with RnBeads. *Nat Methods* 2014; 11: 1138-1140.

8. Lehne B, Drong AW, Loh M, Zhang W, Scott WR, Tan ST, Afzal U, Scott J, Jarvelin MR, Elliott P, McCarthy MI, Kooner JS, Chambers JC. A coherent approach for analysis of the Illumina HumanMethylation450 BeadChip improves data quality and performance in epigenome-wide association studies. *Genome Biol* 2015; 16: 37.

**Table E1.** Estimates of the SNP-BMI association for 97 SNPs from Locke et al. (Nature 2015;518:197-206).

| **SNP** | **Nearest gene** | **EA** | **OA** | **EAF** | **GX** | **SE GX** | **F** |
| --- | --- | --- | --- | --- | --- | --- | --- |
| rs1558902 | *FTO* | A | T | 0.413 | 0.086 | 0.004 | 462 |
| rs6567160 | *MC4R* | C | T | 0.238 | 0.06 | 0.004 | 225 |
| rs13021737 | *TMEM18* | G | A | 0.827 | 0.065 | 0.005 | 169 |
| rs10938397 | *GNPDA2* | G | A | 0.429 | 0.043 | 0.004 | 116 |
| rs543874 | *SEC16B* | G | A | 0.194 | 0.054 | 0.005 | 117 |
| rs2207139 | *TFAP2B* | G | A | 0.174 | 0.046 | 0.005 | 85 |
| rs11030104 | *BDNF* | A | G | 0.789 | 0.039 | 0.005 | 61 |
| rs3101336 | *NEGR1* | C | T | 0.609 | 0.035 | 0.004 | 77 |
| rs7138803 | *BCDIN3D* | A | G | 0.384 | 0.036 | 0.004 | 81 |
| rs10182181 | *ADCY3* | G | A | 0.46 | 0.03 | 0.004 | 56 |
| rs3888190 | *ATP2A1* | A | C | 0.402 | 0.032 | 0.004 | 64 |
| rs1516725 | *ETV5* | C | T | 0.871 | 0.042 | 0.006 | 49 |
| rs12446632 | *GPRC5B* | G | A | 0.866 | 0.043 | 0.006 | 51 |
| rs2287019 | *QPCTL* | C | T | 0.806 | 0.034 | 0.005 | 46 |
| rs16951275 | *MAP2K5* | T | C | 0.781 | 0.033 | 0.004 | 68 |
| rs3817334 | *MTCH2* | T | C | 0.408 | 0.026 | 0.004 | 42 |
| rs2112347 | *POC5* | T | G | 0.633 | 0.024 | 0.004 | 36 |
| rs12566985 | *FPGT-TNNI3K* | G | A | 0.442 | 0.021 | 0.004 | 28 |
| rs3810291 | *ZC3H4* | A | G | 0.665 | 0.031 | 0.004 | 60 |
| rs7141420 | *NRXN3* | T | C | 0.522 | 0.029 | 0.004 | 53 |
| rs13078960 | *CADM2* | G | T | 0.196 | 0.031 | 0.005 | 38 |
| rs10968576 | *LINGO2* | G | A | 0.317 | 0.029 | 0.004 | 53 |
| rs17024393 | *GNAT2* | C | T | 0.037 | 0.067 | 0.011 | 37 |
| rs657452 | *AGBL4* | A | G | 0.392 | 0.022 | 0.004 | 30 |
| rs12429545 | *OLFM4* | A | G | 0.133 | 0.038 | 0.006 | 40 |
| rs12286929 | *CADM1* | G | A | 0.522 | 0.017 | 0.004 | 18 |
| rs13107325 | *SLC39A8* | T | C | 0.071 | 0.052 | 0.008 | 42 |
| rs11165643 | *PTBP2* | T | C | 0.582 | 0.023 | 0.004 | 33 |
| rs7903146 | *TCF7L2* | C | T | 0.713 | 0.016 | 0.004 | 16 |
| rs10132280 | *STXBP6* | C | A | 0.682 | 0.02 | 0.004 | 25 |
| rs17405819 | *HNF4G* | T | C | 0.7 | 0.027 | 0.004 | 46 |
| rs6091540 | *ZFP64* | C | T | 0.719 | 0.021 | 0.004 | 28 |
| rs1016287 | *LINC01122* | T | C | 0.286 | 0.028 | 0.004 | 49 |
| rs4256980 | *TRIM66* | G | C | 0.644 | 0.023 | 0.004 | 33 |
| rs17094222 | *HIF1AN* | C | T | 0.209 | 0.025 | 0.005 | 25 |
| rs12401738 | *FUBP1* | A | G | 0.353 | 0.022 | 0.004 | 30 |
| rs7599312 | *ERBB4* | G | A | 0.724 | 0.017 | 0.004 | 18 |
| rs2365389 | *FHIT* | C | T | 0.581 | 0.021 | 0.004 | 28 |
| rs205262 | *C6orf106* | G | A | 0.27 | 0.022 | 0.004 | 30 |
| rs2820292 | *NAV1* | C | A | 0.557 | 0.02 | 0.004 | 25 |
| rs12885454 | *PRKD1* | C | A | 0.639 | 0.018 | 0.004 | 20 |
| rs9641123 | *CALCR* | C | G | 0.43 | 0.029 | 0.005 | 34 |
| rs12016871 | *MTIF3* | T | C | 0.203 | 0.03 | 0.005 | 36 |
| rs16851483 | *RASA2* | T | G | 0.066 | 0.056 | 0.009 | 39 |
| rs1167827 | *HIP1* | G | A | 0.559 | 0.023 | 0.004 | 33 |
| rs758747 | *NLRC3* | T | C | 0.266 | 0.023 | 0.004 | 33 |
| rs1928295 | *TLR4* | T | C | 0.549 | 0.021 | 0.004 | 28 |
| rs9925964 | *KAT8* | A | G | 0.62 | 0.018 | 0.004 | 20 |
| rs11126666 | *KCNK3* | A | G | 0.278 | 0.015 | 0.004 | 14 |
| rs2650492 | *SBK1* | A | G | 0.304 | 0.021 | 0.004 | 28 |
| rs6804842 | *RARB* | G | A | 0.579 | 0.02 | 0.004 | 25 |
| rs12940622 | *RPTOR* | G | A | 0.57 | 0.015 | 0.004 | 14 |
| rs7164727 | *LOC100287559* | T | C | 0.688 | 0.015 | 0.004 | 14 |
| rs11847697 | *PRKD1* | T | C | 0.043 | 0.051 | 0.01 | 26 |
| rs4740619 | *C9orf93* | T | C | 0.544 | 0.017 | 0.004 | 18 |
| rs492400 | *USP37* | C | T | 0.425 | 0.016 | 0.004 | 16 |
| rs13191362 | *PARK2* | A | G | 0.876 | 0.032 | 0.006 | 28 |
| rs3736485 | *DMXL2* | A | G | 0.455 | 0.016 | 0.004 | 16 |
| rs17001654 | *SCARB2* | G | C | 0.154 | 0.029 | 0.006 | 23 |
| rs11191560 | *NT5C2* | C | T | 0.09 | 0.027 | 0.006 | 20 |
| rs2080454 | *CBLN1* | C | A | 0.4 | 0.017 | 0.004 | 18 |
| rs7715256 | *GALNT10* | G | T | 0.42 | 0.017 | 0.004 | 18 |
| rs2176040 | *LOC646736* | A | G | 0.37 | 0.012 | 0.004 | 9 |
| rs1528435 | *UBE2E3* | T | C | 0.632 | 0.02 | 0.004 | 25 |
| rs2075650 | *TOMM40* | A | G | 0.85 | 0.022 | 0.006 | 13 |
| rs1000940 | *RABEP1* | G | A | 0.316 | 0.021 | 0.004 | 28 |
| rs2033529 | *TDRG1* | G | A | 0.288 | 0.018 | 0.004 | 20 |
| rs11583200 | *ELAVL4* | C | T | 0.392 | 0.02 | 0.004 | 25 |
| rs7239883 | *LOC284260* | G | A | 0.388 | 0.021 | 0.004 | 28 |
| rs2836754 | *ETS2* | C | T | 0.62 | 0.013 | 0.004 | 11 |
| rs9400239 | *FOXO3* | C | T | 0.693 | 0.02 | 0.004 | 25 |
| rs10733682 | *LMX1B* | A | G | 0.474 | 0.018 | 0.004 | 20 |
| rs11688816 | *EHBP1* | G | A | 0.525 | 0.02 | 0.004 | 25 |
| rs11057405 | *CLIP1* | G | A | 0.902 | 0.026 | 0.007 | 14 |
| rs9914578 | *SMG6* | G | C | 0.209 | 0.015 | 0.005 | 9 |
| rs977747 | *TAL1* | T | G | 0.392 | 0.018 | 0.004 | 20 |
| rs2121279 | *LRP1B* | T | C | 0.147 | 0.025 | 0.005 | 25 |
| rs29941 | *KCTD15* | G | A | 0.671 | 0.021 | 0.004 | 28 |
| rs11727676 | *HHIP* | T | C | 0.912 | 0.027 | 0.008 | 11 |
| rs3849570 | *GBE1* | A | C | 0.357 | 0.016 | 0.004 | 16 |
| rs9374842 | *LOC285762* | T | C | 0.744 | 0.023 | 0.004 | 33 |
| rs6477694 | *EPB41L4B* | C | T | 0.364 | 0.016 | 0.004 | 16 |
| rs4787491 | *INO80E* | G | A | 0.51 | 0.022 | 0.004 | 30 |
| rs1441264 | *MIR548A2* | A | G | 0.604 | 0.019 | 0.004 | 23 |
| rs7899106 | *GRID1* | G | A | 0.053 | 0.047 | 0.008 | 35 |
| rs2176598 | *HSD17B12* | T | C | 0.249 | 0.022 | 0.004 | 30 |
| rs2245368 | *PMS2L11* | C | T | 0.178 | 0.03 | 0.007 | 18 |
| rs17203016 | *CREB1* | G | A | 0.196 | 0.022 | 0.005 | 19 |
| rs17724992 | *PGPEP1* | A | G | 0.744 | 0.02 | 0.004 | 25 |
| rs7243357 | *GRP* | T | G | 0.817 | 0.02 | 0.005 | 16 |
| rs16907751 | *ZBTB10* | C | T | 0.918 | 0.04 | 0.008 | 25 |
| rs1808579 | *C18orf8* | C | T | 0.535 | 0.02 | 0.004 | 25 |
| rs13201877 | *IFNGR1* | G | A | 0.141 | 0.021 | 0.005 | 18 |
| rs2033732 | *RALYL* | C | T | 0.747 | 0.017 | 0.004 | 18 |
| rs9540493 | *MIR548X2* | A | G | 0.452 | 0.021 | 0.004 | 28 |
| rs1460676 | *FIGN* | C | T | 0.172 | 0.024 | 0.005 | 23 |
| rs6465468 | *ASB4* | T | G | 0.307 | 0.019 | 0.004 | 23 |

EA, effect allele. OA, other allele. EAF, effect allele frequency. GX, per-allele genetic effect on body mass index. SE GX, standard error of GX. F, F statistic.

**Table E2.** Association of CpG methylation with the weighted genetic risk score for BMI in ECRHS and NFBC (screening phase).

| **CpG** | **Gene** | **Position*** | **Beta (SE)****  **(ECRHS; n = 470)** | **Beta (SE)****  **(NFBC; n = 681)** | **Meta-*P*** |
| --- | --- | --- | --- | --- | --- |
| cg09046979 | *SBK1* | 16:28333134 | -0.61 (0.14) | -0.003 (0.0007) | 1.1x10^-8^ |
| cg12580248 | *NPIPB11* | 16:29412940 | 0.58 (0.12) | 0.002 (0.0005) | 3.7x10^-10^ |

*Chromosomal position. **Beta coefﬁcient and standard error (SE); the beta coefﬁcient is to be interpreted as the change in methylation per minor-allele count unit increase.

**Table E3.** Independent cis-SNPs selected (from online mQTL database) as instrumental variable for genetic risk score for BMI-responsive CpG methylation in the 2nd-step MR.

| **CpG** | **Gene** | **Chr** | **SNP** | **EA** | **OA** | **EAF** | **GX** | **SE GX** | ***P*** | **F** |
| --- | --- | --- | --- | --- | --- | --- | --- | --- | --- | --- |
| cg09046979 | *SBK1* | 16 | rs9938394 | C | T | 0.24 | 0.30 | 0.05 | 7.7x10^-9^ | 34 |
|  |  |  | rs9939450 | C | T | 0.49 | 0.48 | 0.04 | 4.4x10^-27^ | 126 |

EA, effect allele. OA, other allele. EAF, effect allele frequency. GX, per-allele genetic effect on DNA methylation. SE GX, standard error of GX. F, F statistic.
